# Hormonal Influence on Breast Cancer Progression

**DOI:** 10.3390/ijms272110002

**Published:** 2026-11-08

**Authors:** Kamalendu De, Gloria M. Calaf, Malabendu Jana, Bhabadeb Chowdhury, Leodan A. Crispin, Debasish Roy

**Affiliations:** 1 Department of Biological Sciences, Midnapore City College, Midnapore 721129, West Bengal, India; kamalendude530@gmail.com; 2 Instituto de Alta Investigación, Universidad de Tarapacá, Arica 1000000, Chile; kcrispi@gestion.uta.cl; 3 Department of Neurological Science, Rush University School of Medicine, Chicago, IL 60612, USA; malabendu_jana@rush.edu; 4 HIV Dynamics and Replication Program, National Institutes of Health, Frederick, MD 21702, USA; bhabadeb.chowdhury@nih.gov; 5 Department of Natural Sciences, Hostos College of The City University of New York, Bronx, NY 10451, USA; droy@hostos.cuny.edu

**Keywords:** breast cancer, cellular mechanism, estrogen, progesterone, insulin, thyroid, adrenal, receptors, tumor microenvironment

## Abstract

Breast cancer remains a leading cause of cancer-related mortality among women worldwide. Beyond classical estrogen receptor-alpha (ERα) and progesterone receptor (PR) signaling, tumor progression, invasion, and treatment resistance are increasingly linked to an interconnected endocrine–metabolic network involving insulin, insulin-like growth factors (IGF-1/IGF-2), thyroid hormones (T3/T4), catecholamines, adrenal steroids, prolactin, and growth hormone within the tumor microenvironment (TME). Following systematic searches of PubMed, Web of Science, and Scopus through September 2025, 141 primary and translational studies were evaluated in this narrative review. Evidence was categorized according to experimental context: in vitro models, animal xenografts and patient-derived xenografts, and human clinical or epidemiological studies. Endocrine–metabolic resistance was characterized as a mechanism through which tumor cells may circumvent ER/HER2-targeted therapies via hyperinsulinemia-associated insulin receptor/IGF-1R signaling, aerobic glycolysis, thyroid hormone-mediated receptor phosphorylation, and sympathetic/adrenergic remodeling of the TME. Hypoxia was associated with radioresistance, whereas reoxygenation enhanced radiosensitivity; trastuzumab emtansine (T-DM1) was recognized as having clinically relevant toxicities, including thrombocytopenia and hepatotoxicity; non-canonical progesterone signaling was considered in PR-negative and triple-negative breast cancer phenotypes; and PR downregulation was associated with anti-estrogen treatment. Resistance to single-receptor-targeted therapies may be promoted by redundant metabolic and neuroendocrine signaling. Improved therapeutic outcomes may require biomarker-guided combination strategies incorporating selective estrogen receptor degraders, CDK4/6, AKT, and PI3Kα inhibitors, antibody–drug conjugates, and metabolic interventions, including metformin and glycemic control.

## 1. Introduction

Breast cancer represents a heterogeneous spectrum of malignancies driven by genetic alterations, epigenetic modifications, and systemic endocrine–metabolic signals [1]. According to GLOBOCAN 2024, female breast cancer was the leading cause of cancer death among women worldwide and the fourth leading cause of cancer death overall, with an estimated 694,000 deaths globally [2]. Historically, the clinical management of hormone-dependent breast cancer has centered on evaluating classical nuclear steroid receptors, specifically estrogen receptor-alpha (ERα) and progesterone receptor (PR), to guide endocrine interventions such as selective estrogen receptor modulators (SERMs), selective estrogen receptor degraders (SERDs), and aromatase inhibitors (AIs) [3,4,5,6].

Advances in high-throughput transcriptomic profiling have refined this classical taxonomy, establishing five intrinsic molecular subtypes: luminal A, luminal B, HER2-enriched, basal-like, and normal breast-like [3,4,7,8,9,10]. This transcriptomic stratification highlights fundamental biological differences between ER-positive (ER+) and ER-negative (ER−) tumors, demonstrating that ER+ malignancies possess distinct luminal gene expression patterns that dictate therapeutic responsiveness [7,8]. Within ER− disease, molecular subtyping has resolved longstanding diagnostic ambiguities regarding basal-like cancers and triple-negative breast cancers (TNBC). While TNBC is immunohistochemically defined by the absence of ER, PR, and HER2 overexpression, transcriptomic analyses show that true basal-like tumors exhibit unique basal cytokeratin profiles, epidermal growth factor receptor (EGFR) expression, and DNA repair deficiencies that distinguish them from non-basal TNBC variants [10,11,12,13].

Despite the efficacy of endocrine therapies in ER+ breast cancer, primary (de novo) and acquired resistance remain major clinical challenges and can contribute to disease recurrence in a substantial proportion of patients [6,14,15]. Conventional paradigms attribute endocrine failure primarily to somatic mutations in the *ESR1* (encoding ERα) gene (e.g., Y537S, D538G) or secondary loss of ERα/PR expression [16,17]. However, accumulating experimental and clinical data indicate that receptor-centric models fail to capture the full spectrum of therapeutic escape. Tumors operate within a complex systemic landscape where non-gonadal hormones, including insulin, insulin-like growth factors (IGF-1, IGF-2), thyroid hormones such as triiodothyronine (T3), thyroxine (T4), neuroendocrine stress mediators (norepinephrine, epinephrine), adrenal steroids (glucocorticoids, androgens), and pituitary hormones (prolactin, growth hormone), converge on tumor cells and stroma [18,19,20,21,22,23].

This review provides a fully updated, mechanistically rigorous synthesis of how multi-hormonal crosstalk drives breast cancer progression and therapeutic evasion. It integrates systemic endocrine signals with metabolic reprogramming (the Warburg effect), extracellular matrix (ECM) remodeling, and tumor microenvironment (TME) cellular interactions.

## 2. Data Collection Method

This narrative review synthesized the literature published from database inception to September 2025, identified through MEDLINE (via PubMed), Web of Science, and Scopus. The search strategy utilized targeted Boolean term combinations, including (‘breast cancer’ OR ‘breast carcinoma’) AND (‘endocrine resistance’ OR ‘metabolic crosstalk’) AND (‘estrogen’ OR ‘progesterone’ OR ‘insulin’ OR ‘IGF-1’ OR ‘thyroid hormone’ OR ‘epinephrine’ OR ‘norepinephrine’ OR ‘glucocorticoid’ OR ‘androgen’) AND (‘tumor microenvironment’ OR ‘angiogenesis’ OR ‘epithelial-mesenchymal transition’ OR ‘CDK4/6’ OR ‘SERD’ OR ‘ADC’).

A total of 141 publications were curated, comprising 58 primary experimental research articles, 82 systematic or narrative reviews, and 1 doctoral dissertation. To support rigorous interpretation and minimize uncritical extrapolation, the synthesized evidence was graded according to experimental context: Level I, human clinical-trial data, prospective cohort studies, and validated meta-analyses; Level II, in vivo animal models, patient-derived xenografts, and transgenic mouse studies; Level III, in vitro cell-line experiments, mechanistic cell-culture assays, and biophysical models; and Level IV, in silico computational modeling, bioinformatic prediction, and mechanistic hypotheses.

## 3. Hormonal and Metabolic Signaling Pathways in Breast Cancer

### 3.1. Estrogen Signaling: Genomic, Non-Genomic, and Metabolic Regulation

17β-Estradiol (E2) is the principal physiological estrogen driving luminal breast cancer cell proliferation and survival [5,24]. In premenopausal women, E2 is synthesized primarily by ovarian granulosa cells under gonadotropin control. In postmenopausal women, circulating ovarian E2 drops precipitously; however, local tissue estrogen concentrations remain elevated due to peripheral aromatization of adrenal androgen precursors (androstenedione and testosterone) by the aromatase enzyme (*CYP19A1*) expressed in stromal fibroblasts and mammary adipose tissue [25].

At the cellular level, lipophilic E2 enters target cells by passive diffusion across the lipid membrane and binds to distinct estrogen receptor pools [5,24]. These include (a) the genomic pathway (classical nuclear action): unbound ERα and ERβ reside predominantly in the nucleus and cytoplasm bound to heat shock protein complexes. Upon E2 binding, ER undergoes conformational realignment, dissociates from chaperones, forms stable homodimers or heterodimers, and translocates to nuclear chromatin. The E2-ER complex binds directly to canonical Estrogen Response Elements (EREs; 5′-AGGTCAnnnTGACCT-3′) or interacts indirectly with tethering transcription factors (AP-1, SP-1, NF-κB) to recruit coactivators (SRC/p160 family, p300/CBP) and drive transcription of cyclin D1 (*CCND1*), c-Myc (*MYC*), and anti-apoptotic Bcl-2 (*BCL2*) [5,26,27]; (b) the non-genomic pathway (rapid membrane/cytoplasmic action): a fraction of ERα resides at the plasma membrane localized within caveolae via palmitoylation. Upon E2 engagement, membrane-bound ERα rapidly forms signalosomes with G-protein-coupled estrogen receptor (GPER/GPR30), receptor tyrosine kinases (EGFR, HER2), and Src family kinases. This triggers rapid activation of intracellular second messenger cascades, specifically Phosphatidylinositol 3-Kinase (PI3K)/Protein Kinase B (Akt) and Ras/Raf/MEK/Extracellular Signal-Regulated Kinase (MAPK/ERK) pathways [28,29,30].

Downstream of non-genomic E2 signaling, activated Akt phosphorylates glycogen synthase kinase-3 beta (GSK-3β) at serine 9 (Ser9) [28,30]. Phosphorylation at Ser9 inhibits GSK-3β kinase activity, preventing it from targeted ubiquitination and degradation of oncogenic substrates such as β-catenin, c-Myc, and cyclin D1. This GSK-3β inactivation maintains metabolic flux, promotes cell cycle progression through G1/S transition, and suppresses apoptosis (Level II/III evidence) [28,30].

Endocrine therapy resistance in ER+ breast cancer frequently involves acquired *ESR1* ligand-binding domain mutations (e.g., Y537S, D538G), which stabilize the active agonist conformation of ERα in the absolute absence of estrogen [16,31]. Furthermore, crosstalk between non-genomic E2 signaling and growth factor receptors drives receptor phosphorylation, epigenetic histone modifications, and alternative splice variant production, supporting tumor growth despite treatment with aromatase inhibitors or tamoxifen [6,14,17].

**Figure 1:**
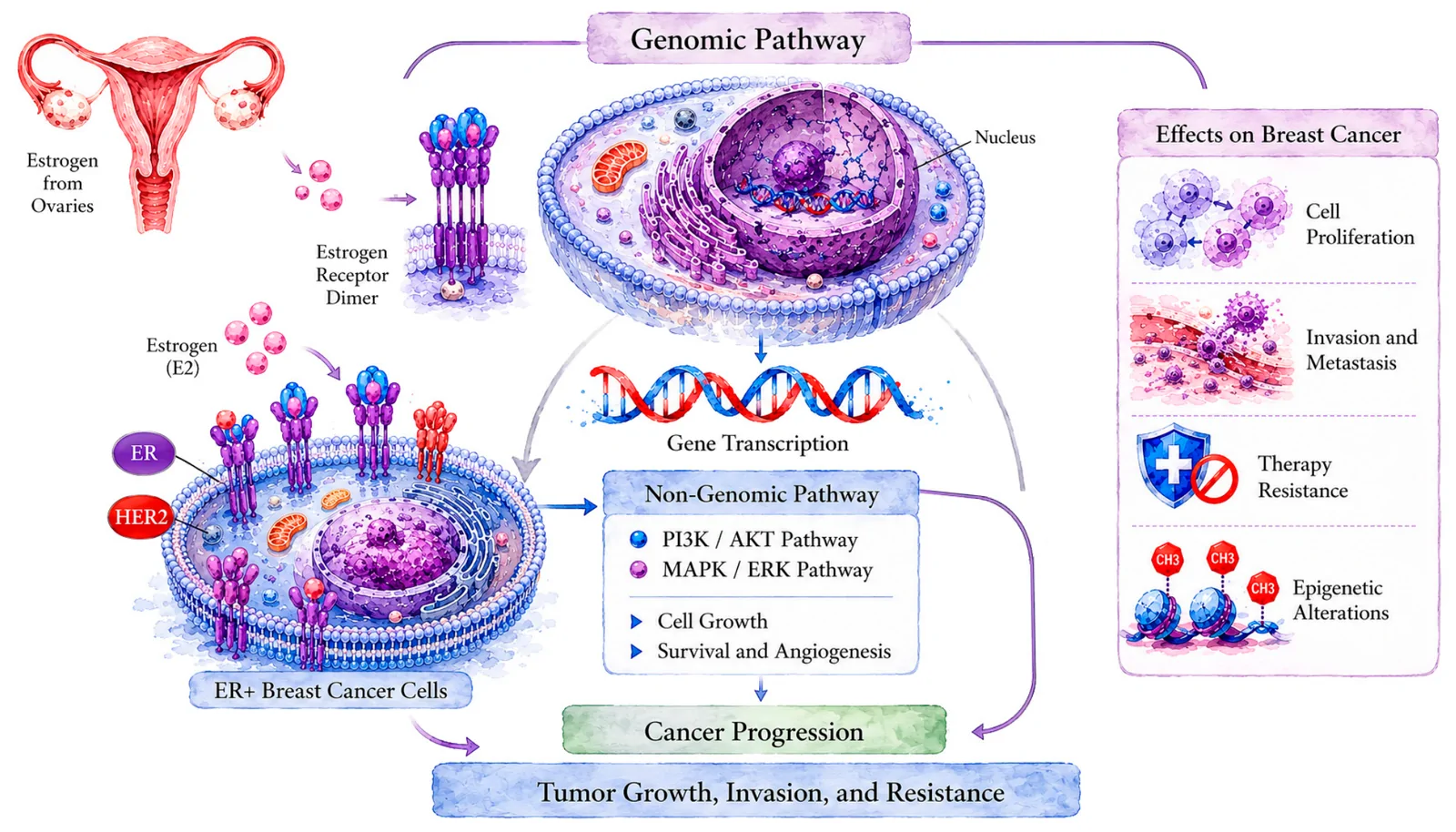
Estrogen-mediated signaling pathways in breast cancer progression. Estrogen (E2) activates estrogen receptors (ERs), initiating genomic signaling through receptor-dependent transcription and non-genomic signaling through membrane-associated receptor complexes. These pathways engage downstream signaling cascades, including PI3K/AKT and MAPK/ERK, that regulate cellular proliferation, survival, angiogenesis, and other processes associated with breast cancer progression and therapeutic resistance. Image created by the authors and visually enhanced with ChatGPT (OpenAI).

Upon binding to estrogen receptors, estrogen activates signaling pathways that regulate cell cycle progression and promote the proliferation and survival of ER+ breast cancer cells (Figure 1). Therefore, treatments targeting estrogen signaling, including aromatase inhibitors and selective estrogen receptor modulators such as tamoxifen, are frequently effective in ER+ tumors, in which estrogen signaling drives growth and proliferation [32].

### 3.2. Progesterone and Estrogen Crosstalk: Receptor Isoforms, Wnt/β-Catenin Signaling, and Endocrine Resistance

Progesterone (P4) acts as a fundamental co-regulator of normal mammary gland development and luminal breast cancer growth [33,34,35]. The biological actions of P4 are mediated through two progesterone receptor isoforms, PR-A (94 kDa) and PR-B (114 kDa), which are encoded by the single *PGR* gene under the control of alternative promoters. In classical estrogen-primed tissue, E2-ERα signaling induces transcriptional upregulation of PR. Consequently, PR expression serves as a canonical functional readout of intact ERα signaling [33,35,36].

Clinical and experimental evidence indicates that endocrine blockade with tamoxifen or aromatase inhibitors reduces ER-driven PR expression [14,35,36]. Loss of PR expression in ER+ breast cancer is recognized as an independent marker of aggressive tumor biology, growth factor hyperactivation, and primary endocrine resistance [36,37]. When ligand-bound PR-B is activated, it can engage intracellular kinase cascades, activating Src/MAPK signaling and modulating transcription of genes regulating tissue remodeling, matrix metalloproteinases (MMPs), and cell–cell adhesion molecules such as E-cadherin [33,34].

Crosstalk between progesterone/estrogen axes and the Wingless-Int1 (Wnt) signaling cascade plays a critical role in expanding breast cancer stem cell (CSC) populations [38,39,40]. Canonical Wnt signaling stabilizes cytoplasmic β-catenin (encoded by *CTNNB1*) by preventing its destruction complex phosphorylation. Stabilized β-catenin translocates to the nucleus, complexing with TCF/LEF transcription factors to drive stemness genes. Paracrine progesterone signaling induces stromal RANKL and *Wnt4* secretion, which stimulates neighboring luminal stem cells and enhances tumor-initiating capacity [38,39,41].

In summary, the interaction between progesterone and estrogen in breast cancer is characterized by reciprocal and complex effects that modify their roles in tumor growth, progression, and response to therapy. Estrogen often primes the tumor for further growth, while progesterone can either promote differentiation or contribute to a more aggressive, undifferentiated phenotype. These hormones act both individually and through reciprocal interactions that influence tumor behavior [33]. Understanding the balance between estrogen and progesterone signaling may inform therapeutic strategies for hormone-responsive breast tumors.

### 3.3. The Insulin and IGF Receptor Family: Isoform Dynamics, Metabolic Reprogramming, and the Warburg Effect

Systemic hyperinsulinemia, metabolic syndrome, and type 2 diabetes are strong clinical predictors of postmenopausal breast cancer incidence and mortality [23,42,43,44]. A critical distinction must be maintained between the insulin receptor (IR) and the insulin-like growth factor 1 receptor (IGF-1R) [45,46,47]. The IR exists as two distinct splice variants, IR-A (lacking exon 11) and IR-B (including exon 11). While IR-B primarily mediates metabolic glucose homeostasis in liver and muscle, tumors preferentially express IR-A, which binds both insulin and IGF-2 with high affinity to drive mitogenic signaling [45,46]. On the other hand, IGF-1R is a tetrameric receptor tyrosine kinase that binds IGF-1 and IGF-2 with high affinity, but binds insulin only at supra-physiological concentrations [45,46]. However, there exist hybrid receptors; i.e., in cancer cells co-expressing IR and IGF-1R, hemireceptors assemble into hybrid IR/IGF-1R complexes that respond primarily to IGF-1 and IGF-2, expanding growth factor responsiveness [46].

Activation of IR-A and IGF-1R recruits Insulin Receptor Substrate 1 and 2 (IRS-1/2), triggering robust PI3K/Akt/mTOR and Ras/Raf/MEK/ERK signaling [48,49]. This axis directly accelerates tumor metabolic reprogramming, enhancing glucose transporter 1 (GLUT1) expression and upregulating key glycolytic enzymes (hexokinase 2, phosphofructokinase 1). This intensifies the Warburg effect, aerobic glycolysis converting glucose to lactate despite ambient oxygen availability [20]. Metformin, an AMPK activator and insulin sensitizer, may attenuate this axis by reducing hepatic gluconeogenesis, lowering circulating insulin levels, and inhibiting mTORC1 signaling in breast-cancer cells (Level I/II evidence) [50,51,52].

**Figure 2:**
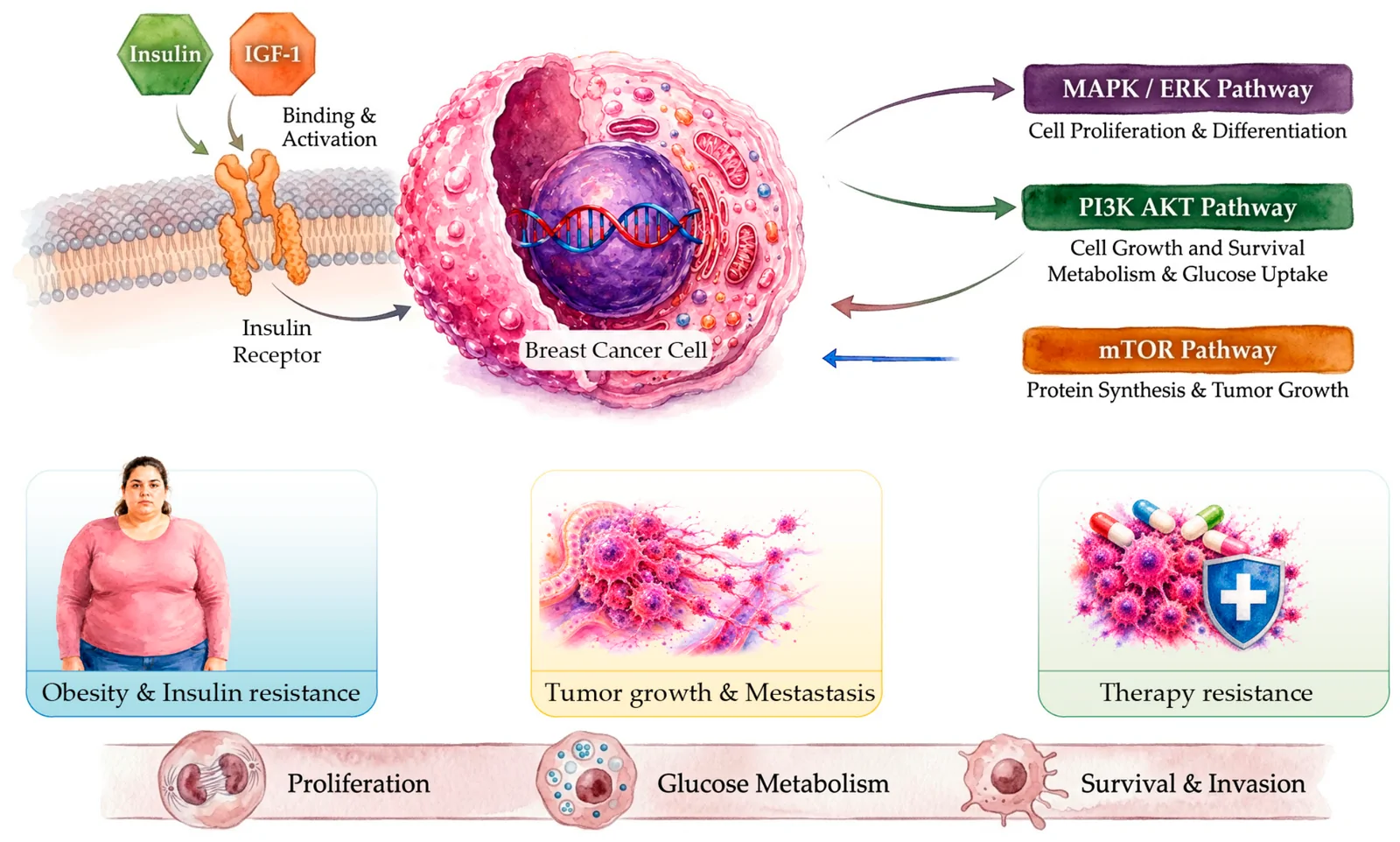
Insulin and insulin-like growth factor signaling in breast cancer. Insulin and insulin-like growth factors activate their respective receptors, including the insulin receptor (IR) and IGF-1 receptor (IGF-1R), engaging downstream PI3K/AKT, MAPK/ERK, and mTOR signaling. These pathways regulate cellular proliferation, survival, and metabolism. Metabolic dysfunction, including insulin resistance, may further influence tumor-promoting signaling, while crosstalk with estrogen receptor pathways can contribute to breast cancer progression and therapeutic resistance. Image created by the authors and visually enhanced with ChatGPT (OpenAI).

The role of insulin and IGF-1 receptor signaling in breast cancer progression is schematically summarized in Figure 2.

In summary, IR signaling regulates cellular metabolism, growth, and survival and may contribute to breast cancer development, progression, metastasis, and therapeutic resistance. Furthermore, insulin-like growth factors (IGFs) amplify the effects of insulin. Insulin promotes cellular proliferation and survival and is involved in the progression of breast cancer. Finally, insulin and IGFs together create a pro-tumorigenic environment that fosters breast cancer progression [46]. Beyond insulin/IGF signaling, thyroid hormones also influence breast-cancer biology. Thyroid hormones, including T3 and T4, are essential regulators of cellular and metabolic activity and have been implicated in cancer biology. Thyroid hormone signaling influences the TME [53]. This TME interacts with several signaling pathways to promote tumor invasion, metastasis, development, and resistance to therapy, and dysregulated thyroid hormone levels can affect breast cancer development. Understanding this interaction can lead to better treatment strategies [53].

### 3.4. Thyroid Hormone Signaling: Receptor, Angiogenesis, Immunomodulation, and Estrogen Crosstalk

Thyroid hormones, such as T3 and T4, regulate systemic cellular basal metabolic rate and interact directly with breast tissue through nuclear thyroid hormone receptors (TRα, encoded by *THRA*; TRβ, encoded by *THRB*) and cell-surface integrin αvβ3 receptors [21,53,54,55,56]. Direct engagement of nuclear TRs and integrin αvβ3 by T3/T4 stimulates pro-angiogenic gene expression, upregulating vascular endothelial growth factor (VEGF) and basic fibroblast growth factor (bFGF) in tumor cells and endothelial compartments (Level II/III evidence) [57,58].

In the TME, thyroid hormone signaling regulates matrix remodeling by inducing matrix metalloproteinases MMP-2 and MMP-9, enhancing extracellular matrix degradation, cellular motility, and epithelial-to-mesenchymal transition (EMT) [56,59,60]. Furthermore, T3/T4 signaling alters immune cell polarization: hyperthyroid states induce M2-like protumoral macrophage polarization and elevate regulatory T cell (Treg) populations, suppressing cytotoxic CD8+ T lymphocyte infiltration (Level II/III evidence) [61,62].

Epidemiological associations between thyroid autoimmunity (e.g., elevated anti-TPO [thyroid peroxidase] antibodies), hypothyroidism, hyperthyroidism, and breast cancer risk remain complex and conflicting in clinical cohorts [63,64]. Mechanistically, T3 enhances ERα transcriptional activity via MAPK-dependent phosphorylation of ERα at Serine-118, demonstrating crosstalk between thyroid axis activity and luminal breast cancer growth [65,66].

### 3.5. Sympathetic and Adrenal Neuroendocrine Signaling: Norepinephrine, Epinephrine, Glucocorticoids, Androgens, and Hypoxia

Chronic physiological and psychological stress stimulates the sympathetic nervous system (SNS) and the hypothalamic–pituitary–adrenal (HPA) axis [67,68,69,70,71]. A crucial physiological distinction must be maintained between sympathetic neural mediators and adrenal hormones. Sympathetic nerve fibers innervate breast tissue and tumor parenchyma, releasing local norepinephrine (NE) into the tumor microenvironment, thereby generating high local concentrations of norepinephrine directly into the TME [22,72]. In physiological stress modeling, locally released NE is the primary driver of intratumoral adrenergic receptor activation [22,71,72]. On the other hand, there is systemic Epinephrine/Adrenaline (E); i.e., epinephrine is synthesized and secreted by the adrenal medulla into the systemic circulation during acute stress responses, acting as an endocrine mediator [70,71].

Breast cancer cells and stromal components express α1-, α2-, β1-, β2-, and β3-adrenergic receptors, with β2-AR being the predominant functional subtype [69,73,74]. Agonist binding to β2-AR couples to G alpha stimulatory subunit (Gαs) proteins, stimulating adenylyl cyclase to elevate intracellular cAMP (cyclic adenosine monophosphate) and activate Protein Kinase A (PKA). PKA activation phosphorylates downstream targets including Src, focal adhesion kinase (FAK), and CREB, driving MMP-2/MMP-9 expression, cell motility, and EMT [22,70,74,75].

Radiotherapy efficacy depends partly on the presence of molecular oxygen: ionizing radiation generates cytotoxic reactive oxygen species (ROS) in oxygenated tissue, contributing to DNA damage, including double-strand breaks [76]. Adrenergic signaling, rapid cell proliferation, and aberrant tumor microvasculature create severe intratumoral hypoxia. Hypoxia suppresses ROS generation and promotes radioresistance; conversely, adequate tumor re-oxygenation enhances radiation-induced ROS formation and radiosensitivity [76].

In addition to adrenergic signaling, the adrenal cortex secretes glucocorticoids (cortisol) and androgen precursors (DHEA, androstenedione) [77,78]. Glucocorticoid receptor (GR) signaling exerts context-dependent effects, suppressing inflammation in early stages but promoting anti-apoptotic signaling, chemoresistance, and metastasis in advanced TNBC [68,78]. Adrenal androgens serve as essential substrates for peripheral aromatization to estrogens in postmenopausal women, while direct Androgen Receptor (AR) activation plays complex, subtype-specific roles in luminal and apocrine breast carcinomas [25,58].

**Figure 3:**
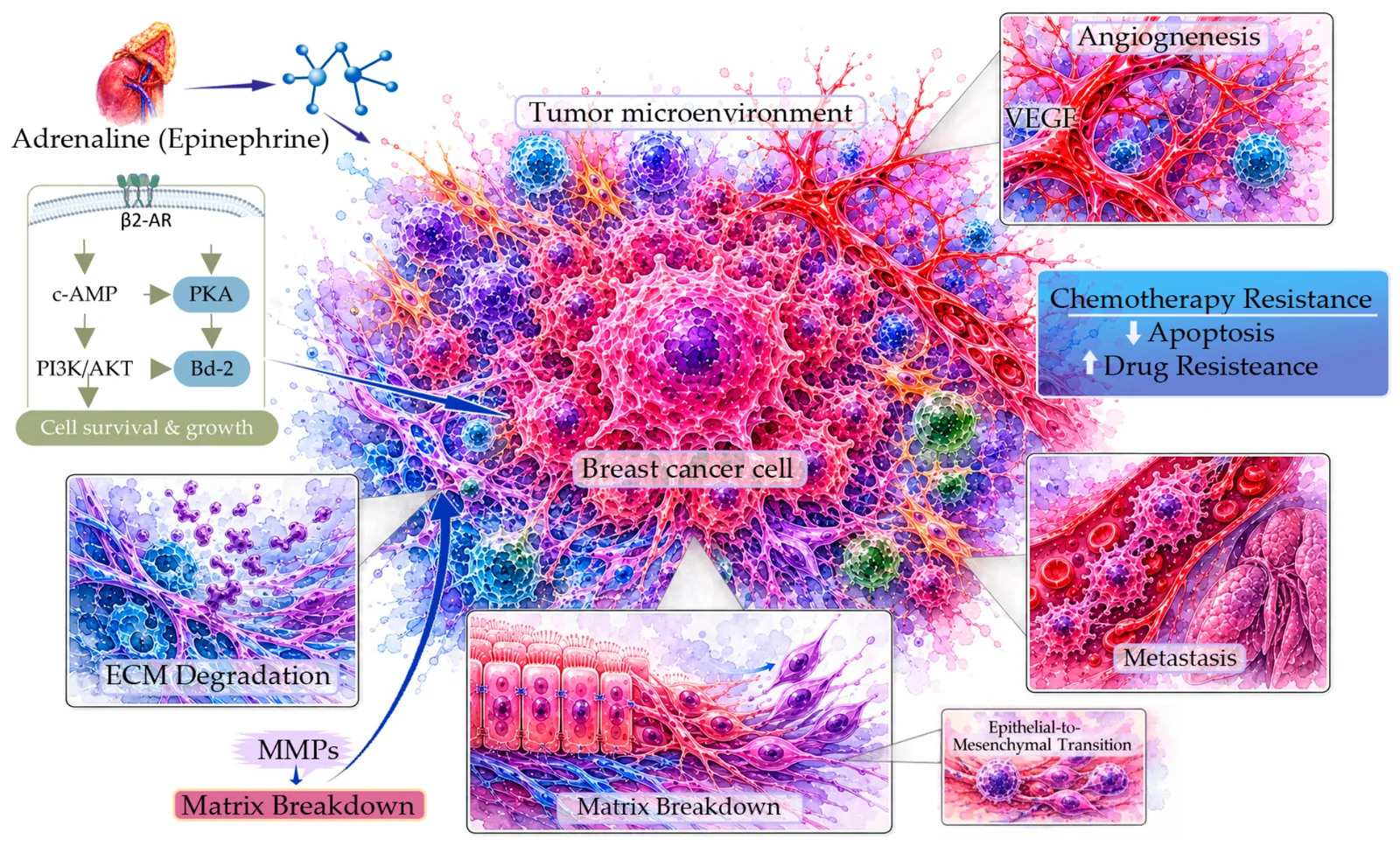
Adrenergic signaling and tumor microenvironment remodeling in breast cancer. Epinephrine activates β2-adrenergic receptors (β2-ARs) on breast cancer cells, engaging downstream cAMP/PKA and PI3K/AKT-associated signaling and promoting cellular survival and growth. Adrenergic signaling may also contribute to angiogenesis, extracellular matrix remodeling, epithelial-to-mesenchymal transition, invasion, metastasis, and treatment resistance. These effects illustrate potential links between neuroendocrine signaling and tumor microenvironment remodeling during breast cancer progression. Image created by the authors and visually enhanced with ChatGPT (OpenAI).

The contribution of adrenaline (epinephrine), a stress hormone secreted by the adrenal glands, to breast cancer progression through adrenergic receptor signaling in the TME is schematically illustrated in Figure 3.

In summary, adrenaline (epinephrine) is produced by the adrenal glands in response to stress, influencing breast cancer progression. It acts through adrenergic receptors present on various cell types, including cancer cells. Adrenaline is released in a stress response, which can change the TME and increase the aggressiveness and development of breast cancer [70]. Research indicates that adrenaline influences the development of tumors, metastasis, angiogenesis, immune evasion, and treatment resistance. Hence, it plays a pivotal role in cancer biology.

### 3.6. Progesterone and Insulin/IGF Interactions: IRS-1 Sensitization and Stemness

Insulin and IGF-1 signaling may modulate sensitivity to progesterone receptor signaling [37,79]. Activation of IGF-1R and IR upregulates PR isoform expression and stabilizes IRS-1 protein levels in luminal breast cancer cells. IRS-1 serves as a molecular scaffold that couples ligand-bound PR-B to downstream PI3K/Akt activation, expanding CD44+/CD24− tumor-initiating breast cancer stem cell populations [37]. In hyperinsulinemic states associated with obesity and metabolic dysfunction, this synergistic interaction accelerates cell cycle progression, enhances VEGF-driven stromal vascularization, and induces endocrine therapy escape (Level II/III evidence) [37,49,80,81,82].

### 3.7. Insulin and Estrogen Interactions: Receptor Crosstalk and Dietary Modulations

Bidirectional positive feedback exists between estrogen and insulin/IGF signaling. E2-ERα signaling upregulates IGF-1R, IR, and IRS-1 expression, enhancing cellular sensitivity to ambient insulin and IGF-1 [42,82,83,84]. In turn, elevated insulin and IGF-1 activate Akt and MAPK/ERK, which directly phosphorylate ERα at Serine-118 and Serine-167, promoting ligand-independent ERα transcriptional activity [48,85].

Emerging evidence from preclinical xenograft models and early clinical investigations indicates that low-carbohydrate, high-fiber, and ketogenic dietary approaches may help improve glycemic control and insulin sensitivity, potentially leading to reductions in fasting insulin levels, modulating IGF-1 bioactivity, reducing inflammatory cytokines, and potentially improving sensitivity to endocrine or PI3K-targeted therapy, suppress IGF-1 bioactivity, decrease systemic inflammatory cytokines (TNF-α, IL-6), and enhance breast tumor sensitivity to tamoxifen, fulvestrant, and PI3K inhibitors (Level II/III evidence) [23,42,79,86].

**Figure 4:**
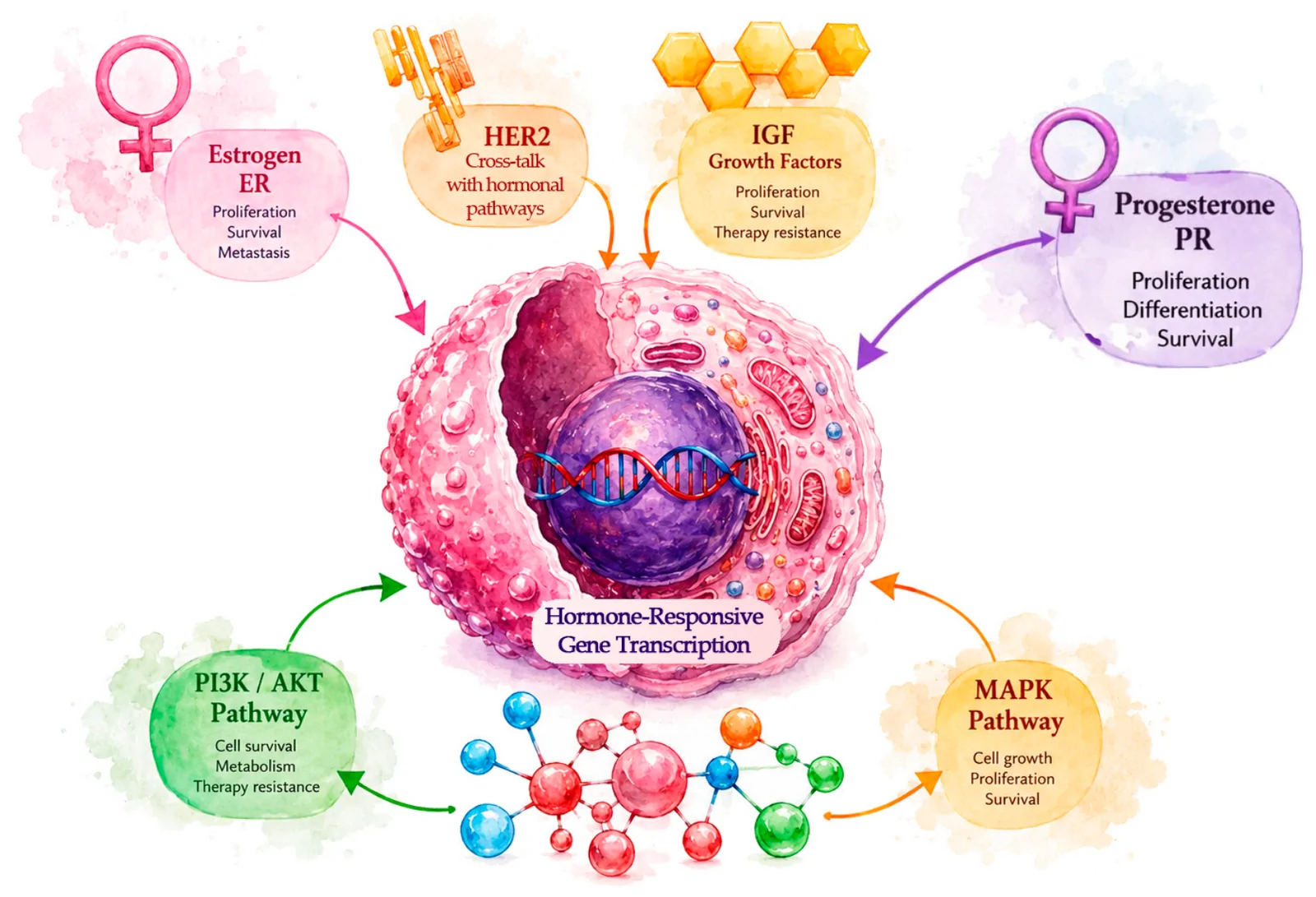
Crosstalk between hormonal and growth-factor signaling pathways in breast cancer. Estrogen and progesterone signaling, mediated by their respective receptors (ER and PR), is integrated with HER2-associated and insulin-like growth factor (IGF)-associated growth-factor signaling. These interconnected networks converge on downstream PI3K/AKT and MAPK/ERK pathways, which regulate hormone-responsive gene transcription and cellular processes associated with breast cancer progression, including proliferation, survival, and metabolic regulation. The figure illustrates how interactions among hormonal and growth-factor signaling pathways may contribute to tumor progression and therapeutic resistance.

The convergence of estrogen, progesterone, and growth-factor signaling through shared intracellular pathways provides an integrated framework for understanding hormonal crosstalk in breast cancer progression (Figure 4).

### 3.8. Thyroid Hormones and Adrenergic Signaling Crosstalk: β2-Adrenergic Receptor Upregulation and Tumor Vascular Effects

A well-documented neuroendocrine interaction occurs between the thyroid axis and the sympathetic nervous system [54,65,70]. Hyperthyroid states elevate tissue responsiveness to catecholamines primarily by transcriptional upregulation of β2-adrenergic receptor density on tumor and cardiovascular cells [54,65]. This elevated β-AR density amplifies catecholamine-mediated cAMP/PKA signaling, driving VEGF expression, ECM degradation, tumor microvascular permeability, and immunomodulatory escape [70,71,72].

β-adrenergic receptor antagonists (e.g., propranolol) mitigate the peripheral downstream sympathoadrenal hyperreactivity induced by elevated thyroid hormone levels [65,87]. Combined pharmacological blockade of β-adrenergic signaling and thyroid-hormone-mediated pathways represents a potential translational strategy for stress-associated, highly vascularized malignancies, but requires further preclinical and clinical evaluation [65,87].

### 3.9. Thyroid Hormones and Estrogen Interactions: Aromatase Induction, SHBG Regulation, and Chromatin Crosstalk

T3/T4 and E2 pathways converge through multiple enzymatic and transcriptional mechanisms [55,65,66]. T3 directly stimulates *CYP19A1* (aromatase) gene expression and enzymatic activity in mammary adipose tissue and stromal cells, elevating local E2 tissue levels [63,65]. Furthermore, thyroid hormone status modifies hepatic synthesis of Sex Hormone-Binding Globulin (SHBG), thereby regulating the bioavailable free fraction of circulating estradiol [55]. At the chromatin level, TR and ERα can bind overlapping hormone response elements or compete for shared nuclear coactivators (SRC-1, p300/CBP), modulating luminal gene transcription [66,88].

### 3.10. Thyroid Hormones and Progesterone Interactions: The Role of Prolactin

Thyroid dysfunction profoundly impacts progesterone signaling and reproductive endocrine homeostasis [63,65,89]. In primary hypothyroidism, diminished circulating T3/T4 levels relieve negative feedback on the hypothalamus and pituitary, leading to elevated Thyrotropin-Releasing Hormone (TRH) and Thyroid-Stimulating Hormone (TSH) [65]. TRH directly stimulates pituitary lactotrophs, inducing hyperprolactinemia. Elevated prolactin alters ovarian progesterone secretion and upregulates prolactin receptor (PRLR) signaling in breast parenchyma, which collaborates with PR to promote luminal cell proliferation and lobuloalveolar stem cell expansion [65,90].

### 3.11. Estrogen and Sympathoadrenal Crosstalk: Positive Feedback and Metastatic Dormancy

E2 and adrenergic pathways establish a pro-metastatic positive feedback loop within luminal breast cancer [74,75,91]. E2 increases the transcription of the *ADRB2* gene (encoding β2-AR) through EREs located in the *ADRB2* promoter, leading to increased expression of the β2-AR [91,92]. Reciprocally, β2-AR signaling activates cAMP/PKA and p38 MAPK cascades, which phosphorylate ERα at Serine-118, driving ligand-independent transcription [75]. In distant metastatic niches (e.g., bone marrow, lung), sympathetic nerve reactivation releasing norepinephrine can awaken dormant single ER+ tumor cells from quiescent dormancy, triggering local proliferation, angiogenesis, and overt metastatic recurrence [72,74].

### 3.12. Progesterone and Adrenergic Signaling: Cyclic AMP Coupling, Migration and Invasion

Adrenergic activation elevates intracellular cAMP levels, which enhances PR transcriptional activity and stabilizes PR protein isoforms [35,74]. Simultaneous exposure to progesterone and catecholamines synergistically upregulates focal adhesion turnover, invadopodia formation, and matrix metalloproteinase secretion, accelerating cellular migration through dense collagen matrices (Level II/III evidence) [22,35,74].

### 3.13. Human Epidermal Growth Factor Receptor 2 (HER2) Signaling, Non-Canonical TNBC Mechanisms, and the Modern Treatment Landscape

HER2 (encoded by *ERBB2* on chromosome 17q12) is a transmembrane receptor tyrosine kinase amplified or overexpressed in 15–20% of invasive breast carcinomas [93,94]. HER2 lacks a known high-affinity ligand; instead, it exists in an open, sterically active conformation that preferentially heterodimerizes with HER3 (*ERBB3*) or EGFR [93,95,96]. HER2-HER3 heterodimers form an exceptionally potent oncogenic signaling unit, driving intense downstream PI3K/Akt/mTOR and Ras/Raf/MEK/ERK activation [96,97].

Ado-trastuzumab emtansine (T-DM1) consists of trastuzumab conjugated to the microtubule inhibitor DM1 via a stable thioether linker [98]. T-DM1 exhibits well-established, dose-limiting toxicities in human clinical trials, including severe grade 3/4 thrombocytopenia (due to megakaryocyte uptake), hepatotoxicity (elevated transaminases, nodular regenerative hyperplasia), peripheral neuropathy, and left ventricular cardiac dysfunction (Level I clinical evidence) [98,99].

Furthermore, biological statements regarding progesterone in TNBC require explicit biochemical qualification. By clinical definition, TNBC lacks nuclear estrogen receptor (ERα < 1%) and nuclear progesterone receptor (PR < 1%) [26,100,101]. Progesterone effects in TNBC models operate through non-canonical pathways, specifically membrane progesterone receptors (mPRα, mPRβ, mPRγ; PAQR family) or Progesterone Receptor Membrane Component 1 (PGRMC1), which couple to G-proteins or EGFR to alter cell motility (Level III evidence) [100,102,103].

The contemporary breast-cancer treatment landscape incorporates advanced molecularly targeted agents [15,99,104,105], such as (i) CDK4/6 inhibitors: palbociclib, ribociclib, and abemaciclib prevent retinoblastoma protein (Rb) phosphorylation, enforcing G1 cell cycle arrest in ER+/HER2− disease [106,107]; (ii) novel oral SERDs: elacestrant (approved for *ESR1*-mutated advanced ER+/HER2− disease following progression on endocrine therapy plus CDK4/6 inhibitors) and camizestrant degrade mutant ERα [31]; (iii) PI3K/AKT pathway inhibitors: alpelisib (alpha-specific PI3K inhibitor for *PIK3CA*-mutated disease), inavolisib (potent PI3Kα inhibitor and degrader), and capivasertib (pan-AKT inhibitor for tumors harboring *PIK3CA*/*AKT1*/*PTEN* alterations) overcome hyperactivated metabolic kinase signaling [97,105,108]; and (iv) next-generation ADCs and HER2-Low Status: trastuzumab deruxtecan (T-DXd; a HER2-directed ADC carrying a topoisomerase I inhibitor payload with a cleavable linker) has revolutionized therapy in both HER2-positive (IHC 3+) and HER2-low (IHC 1+ or IHC 2+/FISH−) advanced breast cancer (Level I clinical evidence) [98,99].

**Figure 5:**
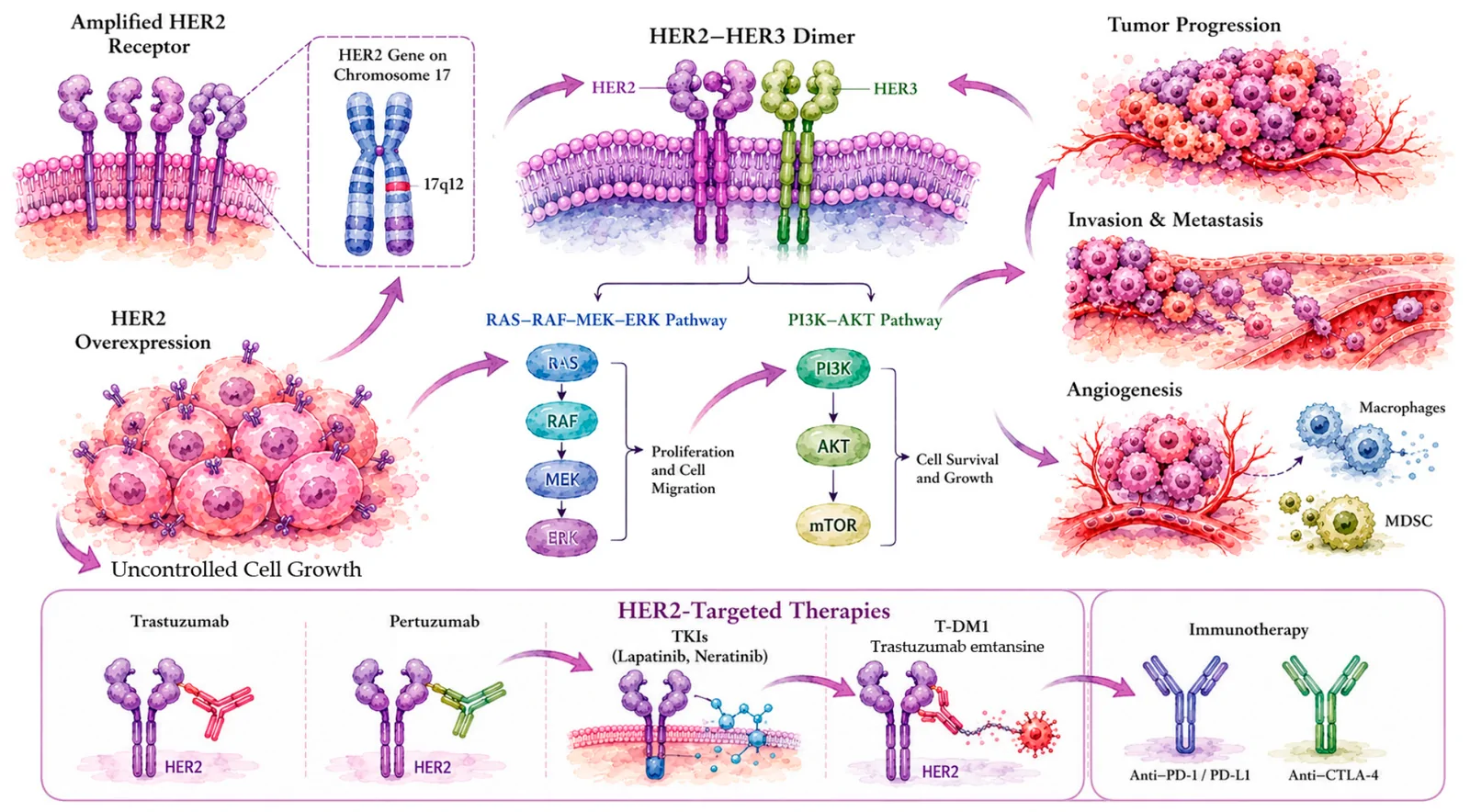
HER2 amplification, downstream signaling, and therapeutic strategies in breast cancer. Amplification of the *ERBB2* gene can lead to HER2 overexpression and receptor dimerization, particularly with HER3, activating downstream RAS–RAF–MEK–ERK and PI3K–AKT–mTOR signaling pathways associated with tumor cell proliferation, survival, migration, and angiogenesis. Interactions between HER2-associated signaling and the tumor microenvironment may further support tumor progression and vascular remodeling. The therapeutic approaches illustrated include HER2-directed monoclonal antibodies (trastuzumab and pertuzumab), tyrosine kinase inhibitors (lapatinib and neratinib), and the antibody–drug conjugate trastuzumab emtansine (T-DM1). Immune checkpoint blockade targeting PD-1/PD-L1 or CTLA-4 is depicted separately as an immunotherapeutic strategy used in selected breast cancer settings. Image created by the authors and visually enhanced with ChatGPT (OpenAI).

Figure 5 presents a schematic representation of the signaling pathways activated by HER2 amplification and their role in promoting breast cancer progression.

## 4. Integrated Endocrine–Metabolic Framework, Microenvironment, and Biomarkers

### 4.1. Definition of Endocrine–Metabolic Resistance

Endocrine–metabolic resistance can be defined as a dynamic state in which breast-cancer cells evade pharmacological inhibition of canonical steroid receptors (ERα and PR) or growth-factor receptors (HER2) by becoming increasingly dependent on systemic hyperinsulinemia, elevated bioavailable IGF-1/2, aerobic glycolysis (Warburg effect), thyroid hormone-induced receptor phosphorylation, and sympathoadrenal/adrenergic TME remodeling [20,23,71,79,109]. Unlike classic endocrine resistance mediated solely by *ESR1* mutations or receptor loss, endocrine–metabolic resistance involves active stromal, metabolic, and neuroendocrine integration that maintains downstream PI3K/Akt/mTOR and MAPK survival signals.

### 4.2. Tumor Microenvironment Compartments: CAFs, TAMs, and Extracellular Stiffness

The TME actively regulates endocrine response through distinct cellular and matrix compartments [110,111]: (i) Cancer-Associated Fibroblasts (CAFs), which secrete hepatocyte growth factor (HGF), CXCL12, and FGF2, which engage *MET* and *FGFR1* on tumor cells to reactivate MAPK/ERK signaling during estrogen deprivation [80,110,112]. Reciprocally, E2 and progesterone modulate CAF lipid metabolism and collagen deposition. (ii) Tumor-Associated Macrophages (TAMs): Rather than adhering to a rigid M1/M2 binary dichotomy, TAMs exist across a fluid functional spectrum [61,109,111]. High tumor lactate generated by Warburg glycolysis stimulates HIF-1α and STAT3 in TAMs, driving secretion of VEGF, IL-10, and TGF-β, which suppresses cytotoxic T-cell surveillance and induces tamoxifen resistance [109,111]; and (iii) ECM mechanics and collagen stiffness. MMPs regulated by thyroid and adrenergic signaling alter collagen cross-linking. Increased matrix stiffness activates mechanosensitive integrin/FAK complexes, reinforcing ligand-independent Akt survival signaling [60,71,110].

### 4.3. Age-Related Hormonal Variations and Histological Subtype Differences

Endocrine–metabolic crosstalk varies across age groups and menopausal states. In premenopausal patients, cyclic ovarian estradiol and progesterone predominate; ovarian suppression with luteinizing hormone-releasing hormone agonists, such as goserelin, may be used to reduce ovarian steroid production [35,113]. In postmenopausal patients, ovarian steroidogenesis ceases, but peripheral aromatization in expanding visceral/mammary adipose tissue maintains tissue E2 levels [25]. Age-related adrenal senescence (adrenopause) alters the DHEA/cortisol ratio, while age-associated increases in visceral adiposity, HOMA-IR, and chronic low-grade inflammation (inflammaging) exacerbate insulin-driven resistance [43,78,79]. Extended pathology spectrum: In invasive lobular carcinoma (ILC), loss of E-cadherin (encoded by *CDH1*) may alter membrane ERα trafficking and enhance sensitivity to IGF-1R signaling, whereas invasive ductal carcinoma (IDC) exhibits higher rates of HER2 amplification and *PIK3CA* mutations [4,10,101].

### 4.4. Biomarker Categorization: Prognostic vs. Predictive vs. Pharmacodynamic

To enable precise translational application, potential biomarkers within this endocrine–metabolic framework must be strictly categorized: (1) prognostic biomarkers (reflect overall clinical outcome regardless of specific therapy): baseline HOMA-IR, elevated serum C-peptide, high BMI (>30 kg/m^2^), low adiponectin/leptin ratio, and stromal TIL density [23,42,79]. (2) Candidate biomarkers indicate differential benefit from a specific targeted intervention. Examples include *ESR1* mutations (Y537S or D538G), which may predict greater benefit from elacestrant than from standard aromatase inhibitors [31]; *PIK3CA* catalytic mutations (E542K, E545K, H1047R), which predict benefit from alpelisib or inavolisib [105,108]; *AKT1* E17K mutation or *PTEN* loss, which predict benefit from capivasertib [97]; HER2-low status (IHC 1+ or IHC 2+/FISH−), which predicts benefit from trastuzumab deruxtecan (T-DXd) [98,99]; and fasting hyperinsulinemia/high IGF-1, which predicts potential benefit from adding metformin or lifestyle glycemic interventions to endocrine therapy [50,51,114]. (3) Pharmacodynamic biomarkers (confirm target engagement in tumor/tissue): Reduction in Ki-67 proliferation index, decreased phosphorylated Akt (p-Akt Ser473), decreased p-S6, and suppression of serum lactate [30,105,109].

## 5. Limitations and Future Directions

A key limitation is the need for longitudinal multi-omics clinical sampling. Prospective clinical trials should incorporate serial liquid biopsies (ctDNA, circulating metabolites, serum insulin/IGF) and spatial transcriptomics to track the temporal evolution of endocrine–metabolic resistance. Second, rational combination design is needed: preclinical and early-phase clinical trials should prioritize multi-node inhibition, pairing oral SERDs or AIs with PI3Kα/AKT inhibitors, CDK4/6 inhibitors, β2-adrenergic antagonists, or insulin-sensitizing metabolic agents. Finally, standardized biomarker validation is required. Composite biomarker scores integrating *ESR1* and *PIK3CA* mutation status, HER2 expression density, metabolic HOMA-IR, and TME immune signatures should undergo prospective clinical validation before routine diagnostic implementation.

## 6. Conclusions

In conclusion, hormonal regulation of breast cancer progression extends far beyond isolated nuclear estrogen and progesterone receptor activation. Breast cancer cell proliferation, invasion, metastasis, and therapeutic evasion are governed by an integrated endocrine–metabolic network encompassing insulin/IGF signaling, thyroid hormones, sympathoadrenal adrenergic mediators, adrenal steroids, and microenvironmental stromal compartments. Monotherapy directed against single steroid receptors frequently fails due to rapid metabolic adaptation and pathway redundancy under the endocrine–metabolic resistance paradigm.

## Data Availability

The data generated in the present study may be requested from the corresponding author.

## Abbreviations

The following abbreviations are used in this manuscript:

ER	Estrogen Receptor
ERα	Estrogen Receptor alpha
T3	Triiodothyronine
T4	Thyroxine
TME	Tumor microenvironment
E2	17β-Estradiol
PGR	Progesterone Receptor
HER2	Human Epidermal Growth Factor Receptor 2
ERα	Estrogen receptor α
PI3K	Phosphatidylinositol 3-kinase
mTOR	Mammalian target of rapamycin
AKT	Protein kinase B
TNBC	Triple-negative breast cancer
IR	Insulin receptor
IGFs	Insulin-like growth factors
EREs	Estrogen response elements
ERK	Extracellular signal-regulated kinase
GSK-3β	Glycogen Synthase Kinase-3 Beta
MEK	Mitogen-activated protein kinase kinase
VEGF	Vascular endothelial growth factor
EGF	Epidermal growth factor
CDK4/6	Cyclin-dependent kinases
TR	Thyroid hormone receptors
FGF	Fibroblast growth factor
MMPs	Matrix metalloproteinases
EMT	Epithelial-to-mesenchymal transition
ECM	Extracellular matrix
α-ARs	α-adrenergic receptors
β-ARs	β-adrenergic receptors
ROS	Reactive oxygen species
SERMs	Selective estrogen receptor modulators
CSCs	Cancer stem cells
CDKs	Cyclin-dependent kinases
IGF-1R	IGF-1 receptors
SNS	Sympathetic nervous system
HPA	Hypothalamic–pituitary–adrenal
MDSCs	Myeloid-derived suppressor cells
ADCs	Antibody–drug conjugates
*THRA*	Thyroid hormone receptor alpha
*THRB*	Thyroid hormone receptor beta
*ERBB2*	Erb-B2 receptor tyrosine kinase 2
*ERBB3*	Erb-B3 receptor tyrosine kinase 3
*IGF1R*	Insulin-like growth factor 1 receptor
*CDH1*	Cadherin 1
*MAP2K1*	Mitogen-activated protein kinase kinase 1
*PIK3CA*	Phosphatidylinositol-4,5-bisphosphate 3-kinase catalytic subunit alpha
*WNT1*	Wnt family member 1
*AKT1*	AKT serine/threonine kinase 1
*CCND1*	Cyclin D1
*HRAS*	HRas proto-oncogene, GTPase
*MYC*	MYC proto-oncogene, bHLH transcription factor
